# Degradation of Internalized αvβ5 Integrin Is Controlled by uPAR Bound uPA: Effect on β1 Integrin Activity and α-SMA Stress Fiber Assembly

**DOI:** 10.1371/journal.pone.0033915

**Published:** 2012-03-21

**Authors:** Lingyan Wang, Benjamin S. Pedroja, Erin E. Meyers, Angelo L. Garcia, Sally S. Twining, Audrey M. Bernstein

**Affiliations:** 1 Department of Ophthalmology, Mount Sinai School of Medicine, New York, New York, United States of America; 2 Department of Biochemistry, Medical College of Wisconsin, Milwaukee, Wisconsin, United States of America; Ecole Polytechnique Federale de Lausanne, Switzerland

## Abstract

Myofibroblasts (Mfs) that persist in a healing wound promote extracellular matrix (ECM) accumulation and excessive tissue contraction. Increased levels of integrin αvβ5 promote the Mf phenotype and other fibrotic markers. Previously we reported that maintaining uPA (urokinase plasminogen activator) bound to its cell-surface receptor, uPAR prevented TGFβ-induced Mf differentiation. We now demonstrate that uPA/uPAR controls integrin β5 protein levels and in turn, the Mf phenotype. When cell-surface uPA was increased, integrin β5 levels were reduced (61%). In contrast, when uPA/uPAR was silenced, integrin β5 total and cell-surface levels were increased (2–4 fold). Integrin β5 accumulation resulted from a significant decrease in β5 ubiquitination leading to a decrease in the degradation rate of internalized β5. uPA-silencing also induced α-SMA stress fiber organization in cells that were seeded on collagen, increased cell area (1.7 fold), and increased integrin β1 binding to the collagen matrix, with reduced activation of β1. Elevated cell-surface integrin β5 was necessary for these changes after uPA-silencing since blocking αvβ5 function reversed these effects. Our data support a novel mechanism by which downregulation of uPA/uPAR results in increased integrin αvβ5 cell-surface protein levels that regulate the activity of β1 integrins, promoting characteristics of the persistent Mf.

## Introduction

Myofibroblasts (Mfs) promote normal healing and wound closure but eventually die by apoptosis [1]. Deregulation of this process leading to the persistence of Mfs contributes to fibrosis and scarring by overproduction of extracellular matrix (ECM) and excessive tissue contraction. Persistent Mfs participate in an autocrine loop of TGFβ activation that leads to accumulation of ECM and stabilization of the Mf phenotype that promotes fibrotic disease [2], [3], [4].

Several factors are known to regulate differentiation of fibroblast to Mfs. Our current work is focused on the contribution of the uPA pathway to this process. uPA is an extracellular serine protease that binds to its receptor, uPAR, and converts plasminogen into plasmin at the cell-matrix interface. In addition to the generation of plasmin, uPA binding to uPAR stimulates uPAR's interaction with integral membrane proteins, such as integrins, which modulate cytoskeletal organization and cell migration [5]. In our previous work, we showed that maintaining full-length uPAR, consisting of 3 domains, (D1D2D3) on the cell-surface, as opposed to cleaved uPAR (D2D3), prevented Mf differentiation [6]. This finding suggested that proper regulation of uPAR is an important part of the normal differentiation program. Our goal in the current work was to understand the mechanism by which full-length uPAR affects Mf differentiation.

Full-length uPAR binds uPA, integrins, among them αvβ3 and αvβ5 integrins [7], [8], and the matrix molecule vitronectin; cleaved uPAR loses the ability to participate in these interactions [9], [10]. Over-expression of cleaved uPAR (D2D3) in cells that lack endogenous uPAR, led to an increase in integrin-mediated cell adhesion, suggesting that the D1 domain of uPAR plays a role in regulating the adhesive functions of integrins [9]. Enhanced cell adhesion promotes Mf differentiation, as stabilized cell attachment to ECM generates the cellular tension required for assembly of the Mf's characteristic alpha-Smooth Muscle Actin (α-SMA) stress fibers [11], [12]. Thus, our finding that, compared to fibroblasts, Mfs have reduced levels of full-length uPAR [6] suggests that Mf integrins may no longer be regulated by uPAR, leading to greater cell adhesion.

Integrin αvβ5 is integral to the Mf phenotype and related TGFβ activity. αvβ5 expression and TGFβ activity are upregulated in Mfs derived from fibrotic, sclerodermal tissue. Furthermore, treatment of these sclerodermal fibroblasts with anti-αvβ5 antibodies reduces TGFβ activity and Mf differentiation [13], [14]. Finally, a mechanism by which αvβ5 activates TGFβ was recently described wherein αvβ5 binds to the RGD sequence in the LAP-TGFβ and the mechanical force generated by integrin-mediated Mf contraction releases active TGFβ [15]. Together, these data suggest that increased levels of αvβ5 contribute to persistent Mfs and fibrotic disease. Since uPAR cleavage leads to an increase in integrin-mediated cell adhesion, and the cleavage and subsequent downregulation of uPAR correlates with Mf differentiation (a cell phenotype that is dependant upon increased cell adhesion) [11], we investigated whether Mfs are regulated by uPA/uPAR's control of integrin αvβ5. Here we report that uPA bound to uPAR regulates integrin β5 levels; in the absence of uPA, ubiquitination and degradation of internalized β5 is drastically reduced leading to a significant increase of cell-surface β5, which promotes integrin β1 binding to a collagen matrix generating a Mf-like phenotype. Defects in protein degradation play an important role in many diseases [16], [17], [18]. However, protein degradation (ubiquitin) pathways have not been widely considered in generation of Mfs or in the pathogenesis of fibrotic healing in general. Our study may open the way to novel therapeutic approaches for the prevention of fibrotic disease.

## Results

### Integrin αvβ5 expression is uniquely associated with the Mf phenotype

**Figure 1:**
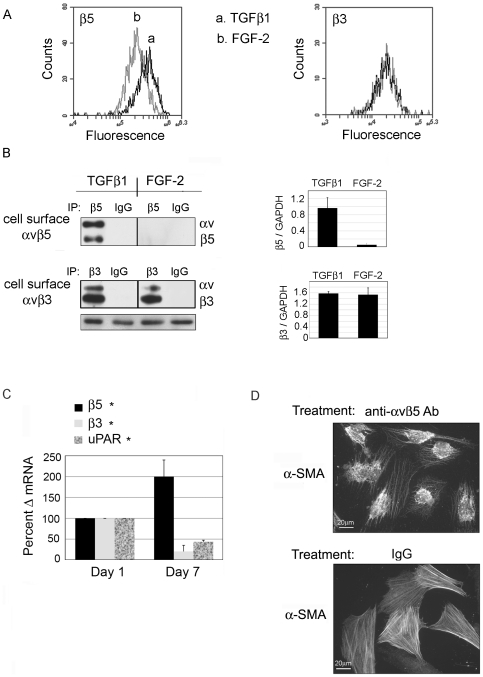
Integrin β5 protein expression is increased on TGFβ-induced Mfs. HCFs were treated with TGFβ1 or FGF-2 plus heparin for 72 hours and analyzed for β5 and β3 cell-surface expression. (A) Flow cytometry (B) Cell-surface biotinylation; lysates were immunoprecipitated (IP) with antibody against integrin β5, separated by SDS-PAGE and probed with streptavidin-HRP to detect biotinylated proteins. Bands were identified by molecular weight. GAPDH indicates equal protein input for IP. Quantification of the β5 band relative to GAPDH input control is shown. (C) Real-time PCR for β5, β3, and uPAR at Day 1 and Day 7 after TGFβ1 treatment. *p value<0.05 for gene expression between Day 1 and Day 7 for each. (D) HCF were stimulated with TGFβ for 48 hours to initiate Mf differentiation and then treated with anti-αvβ5 function blocking antibodies or IgG controls overnight. Cells were fixed and immunostained for α-Smooth Muscle Actin (α-SMA). N = 3–5 for each experiment.

To demonstrate that αvβ5 expression and function is key to maintaining the TGFβ-induced Mf phenotype, we treated human primary corneal fibroblasts (HCFs) with either TGFβ1 or as a comparison, FGF-2 plus heparin a combination that promotes the fibroblast phenotype [19], each for 72 hours prior to analysis. All experiments were performed under defined conditions (supplemented-serum free conditions (SSFM, see methods) on collagen type I). Surface expression of the integrin β5 was assessed in two distinct manners, indirect immunostaining of live cells followed by quantitative flow cytometry or immunoblotting of biotin labeled surface proteins. To provide a comparison to levels of integrin β5, we also measured cell-surface levels of integrin β3. By flow cytometry, the TGFβ1 treatment resulted in a cell-surface integrin β5 level that was 1.56+/−.01 fold higher, *p value<.05 than that observed in the presence of FGF-2 (Figure 1A). Unlike the case for β5, the expression of β3 was not affected by the difference in growth factor exposure (Figure 1A, right). The cell-surface biotinylation approach yielded a more extreme difference in the degree of β5 expression (Figure 1B). As in the flow cytometry case, the difference in growth factor exposure did not affect the amounts of β3. Qualitatively the flow data and cell-surface IP both show that TGFβ1 treatment leads to an increase in cell-surface integrin β5. The observed quantitative difference in integrin expression is most likely caused by the vast difference in methodology; prior to immunoblotting cells have to be solubilized, a process that is likely to recover most of the surface integrins. In contrast, flow cytometry requires that the strongly adherent fibroblasts be first detached. Previous studies have demonstrated that cell detachment leads to the internalization of multiple plasma membrane proteins including cell-surface [20], [21] and thus may reduce cell-surface staining.

### TGFβ induction of β5 expression is time dependent

Our previous work showed that in cultured HCFs treated for 7 days with TGFβ1, uPAR is cleaved into its D2D3 form and by day 7, uPAR protein expression is nearly absent on the resultant Mfs [6]. Since β5 is associated with the TGFβ-induced Mf phenotype we investigated whether an increase in β5 gene expression in response to TGFβ1 treatment correlated with the enhancement of Mf differentiation; integrin β3 and uPAR served as controls. Real time PCR was performed on mRNA isolated at Day 1 and Day 7 of TGFβ1 treatment. While β5 mRNA expression increased by 100% on day 7 of treatment (fully differentiated Mfs), the β3 decreased by 80% and uPAR decreased by 56% (Figure 1C). To assess the importance of β5 to Mf differentiation, we blocked functional interactions of integrin β5 with an antibody and measured the impact of this treatment on α-SMA stress fiber organization, a key marker of the Mf. HCFs were differentiated into Mfs by treating with 1 ng/ml TGFβ1 for 48 hours and then incubated for 24 hours with a blocking anti-αvβ5 antibody. Compared to IgG treated cells, cells treated with anti-αvβ5 disrupted α-SMA stress fibers (Figure 1D), although the number of cells adhering to collagen was not reduced. (The affect of this antibody on cell adhesion was quantified and will be discussed later.) These data demonstrate that, similar to the previously reported effect in sclerodermal fibroblasts [13] blockade of β5 decreases the TGFβ-induced indicators of Mf differentiation. Of significance is that with TGFβ1 treatment, uPAR and β5 have reciprocal expression patterns (Figure 1C). This led us to investigate whether cell-surface uPA and/or uPAR regulates β5 levels. For the remainder of the experiments, TGFβ was not added.

### uPA and uPAR regulate integrin β5 protein levels

**Figure 2:**
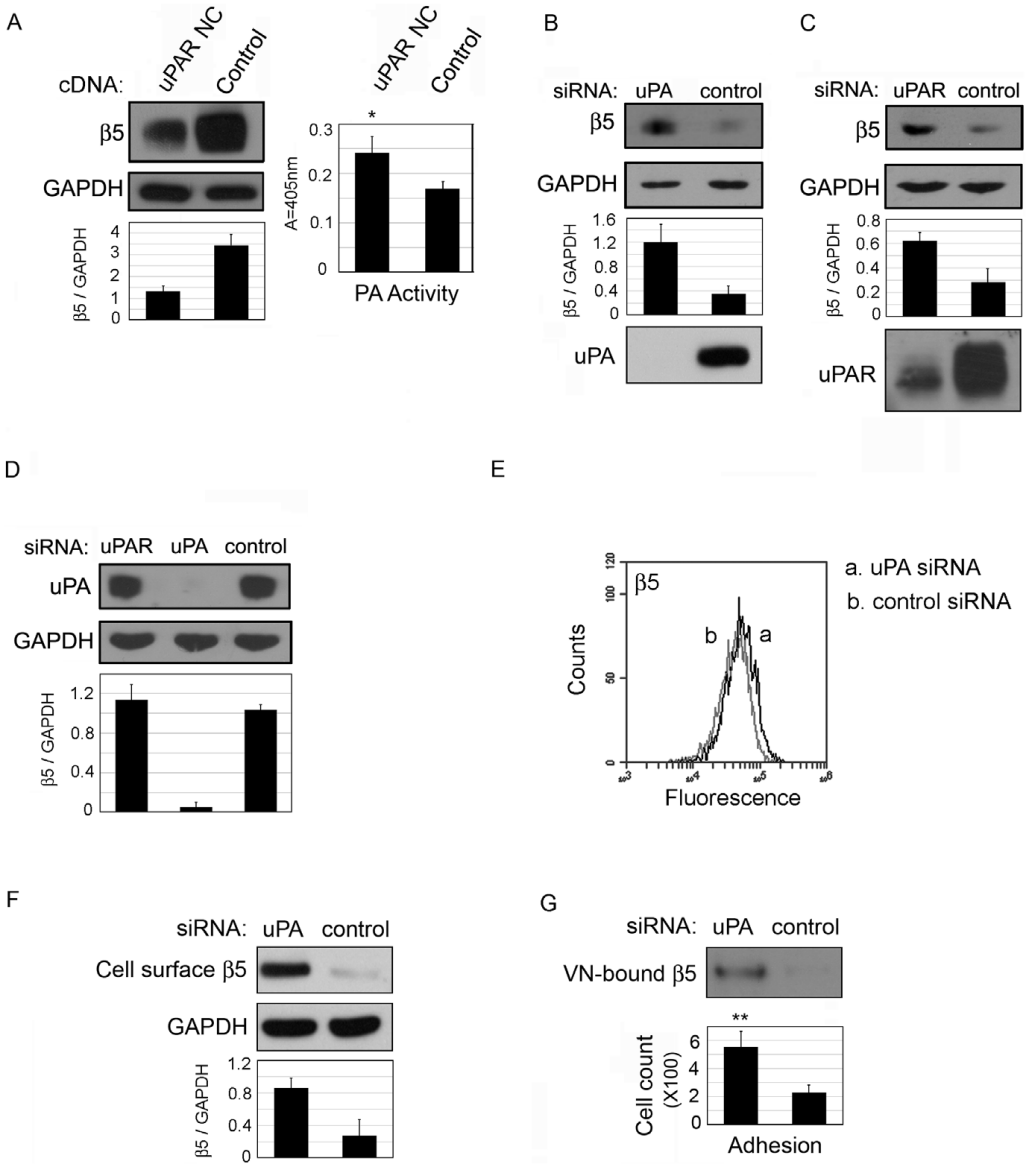
uPA expression controls integrin β5 levels. (A) HCFs were transfected with a non-cleavable uPAR mutant cDNA (uPAR NC) or control vector. After 48 hours, cells were lysed and subjected to Western blot analysis for integrin β5. GAPDH indicates equal loading. Cell associated PA activity was measured colorimetrically by adding plasminogen and a chromogenic substrate for plasmin to cell lysates. Cell associated PA activity is shown to the right. *p value<0.05. HCF were transfected with (B) uPA siRNA or non-targeting siRNA control or (C) uPAR siRNA or non-targeting siRNA control. After 24 hours, lysates were Western blotted for β5 and uPA or uPAR to confirm knockdown. (D) HCF were transfected with uPAR siRNA, uPA siRNA, or control siRNA. After 24 hours, lysates were Western blotted for uPA. The data in (D) demonstrate that uPA is still expressed after uPAR silencing with uPAR siRNA. GAPDH indicates equal loading. (E–G) HCFs were transfected with uPA siRNA or control siRNA. (E) Flow cytometry. (F) Cell-surface biotinylation: After 24 hours, HCFs were cell-surface biotinylated before lysing and IP with streptavidin-HRP beads followed by Western for β5. GAPDH indicates equal input of protein for IP. (G) Integrin β5 bound to vitronectin (VN), top: After 24 hours, HCFs were treated with a cleavable cross-linker and cells were removed by lysis with 0.1% SDS. Next, the cross-linker was cleaved, releasing VN-bound proteins. This fraction was concentrated equal protein was Western blotted for β5. Bottom: Cell adhesion on VN: 24 hours after transfection, cells were detached and plated on VN for another 24 hours. Cells were fixed, stained with DAPI and counted. **p value<0.01. N = 3–7 for each experiment.

Full-length uPAR but not cleaved uPAR on the cell-surface binds to extracelluar uPA. Thus over-expressing a form of uPAR that cannot be cleaved (non-cleavable uPAR) will maintain both full-length uPAR and uPA on the cell-surface. To determine if an increase in uPA bound to uPAR on the cell surface regulates the protein levels of integrin β5, HCFs were transfected with non-cleavable uPAR cDNA (uPAR NC) [22] or vector alone (control). Compared to control, over-expression of uPAR NC (confirmed by real time PCR, data not shown) reduced β5 levels by an average 61% (Figure 2A). uPAR NC expression led to a 43% increase in cell-associated uPA activity, (right of the Western blot in Figure 2A). Thus there was less β5 when uPA was maintained on the cell-surface.

To test the effect of uPA/uPAR downregulation on αvβ5 levels, HCFs were transfected with siRNA to uPA, uPAR or a non-targeting control. siRNA knock-down of uPA and uPAR was confirmed by Western blotting. In cells in which uPA was silenced, the total β5 protein levels were increased from approximately 2 to 4-fold (Figure 2B); (the range presumably reflects the variation in the primary fibroblasts obtained from individual donors). In cells with knocked-down uPAR, the β5 levels were increased by approximately 2-fold (Figure 2C). The fact that uPAR knockdown also lead to an increase in β5 levels suggests that the presence of uPA without uPAR (confirmed in Figure 2D) does not regulate β5. Thus, as would be expected, uPA binding to uPAR appears to be required for its regulation of β5.

Next we tested whether the increase in β5 protein in uPA-silenced cells was reflected in increased cell-surface accumulation of the integrin. Cells were transfected with uPA-siRNA or control siRNA, and 24 hrs later, cells were analyzed by flow cytometry. Here we demonstrate that uPA-silencing increased β5 on the cell-surface by 1.22+/−.07 fold, **p value<.01 (Figure 2E).These data were supported by cell-surface biotinylation. Biotinylated lysates were immunoprecipitated with streptavidin beads followed by Western blot for β5. Results shown in Figure 2F demonstrate that uPA-silencing leads to an average 3.1-fold increase in cell-surface αvβ5 using this technique. Again, similar to Figure 1, cell-surface biotinylation reveals greater differences between samples, likely because the process of detachment for flow cytometry reduces the detection of integrins on the cell surface as stated above. Our demonstration that cell-surface integrin β5 is increased after uPA-silencing is supported by the fact that β5 function is also increased. Specifically, binding of integrin β5 with its primary ligand, vitronectin (VN) and cell adhesion to VN were both significantly increased after uPA-silencing (Figure 2G). After transfection with uPA and control siRNA, cells were seeded on VN for 24 hrs and treated with a cleavable cross-linking reagent, followed by removal of cells with a buffer containing 0.1% SDS. Cell membrane proteins that were cross-linked to matrix were cleaved and equal amounts of protein in each sample were analyzed for β5 content by Western blotting. A lack of GAPDH on Western blots confirmed that cells were not present in the protein fraction that was released from the matrix. As expected, based on the flow cytometry and cell-surface biotinylation experiments, the association of integrin β5 with VN is increased in uPA-silenced cells (Figure 2G, top). Next we performed a typical adhesion assay on VN after uPA-silencing. Cells were transfection with uPA siRNA or control and seeded onto collagen. After 24 hours cells were detached and seeded on VN for 1 hour. In support of our finding that differences by flow cytometry were difficult to observe directly after cell detachment, uPA-silenced cells plated on VN for 1 hour demonstrated only a small increase in cell adhesion to VN (data not shown). However, when the transfected cells were seeded on VN and incubated for 24 hours, cell adhesion on VN, was 2.4-fold higher in uPA-silenced cells than in control (Figure 2G, bottom). Together these experiments on VN reveal that the increased cell surface β5 after uPA-silencing results in a functional increase in ligand binding.

### uPA-mediated degradation of internalized β5 controls β5 protein levels

**Figure 3:**
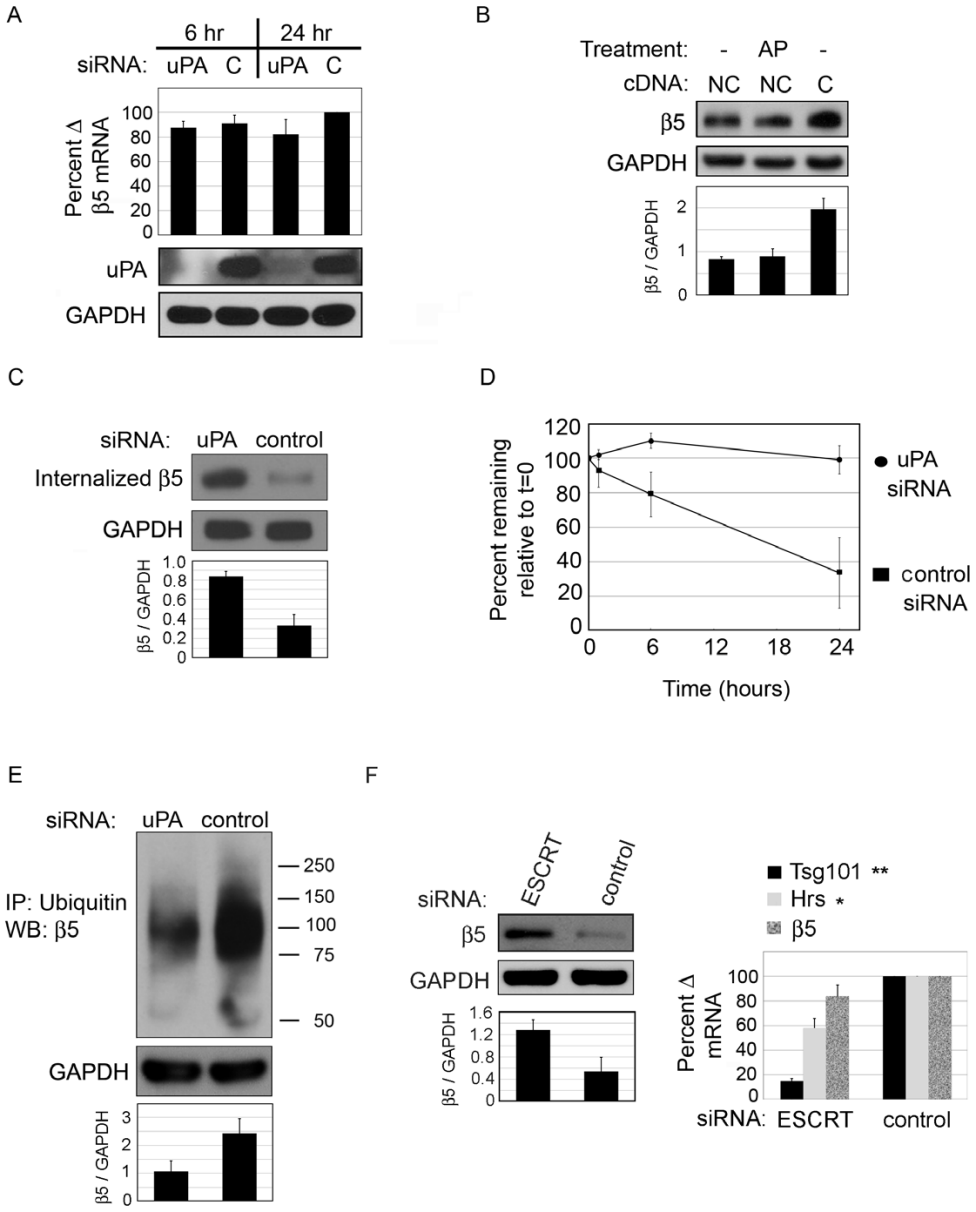
uPA-silencing reduces the degradation of internalized β5. (A) Gene expression: HCFs were transfected with uPA siRNA or control siRNA and lysed for RNA or protein at 6 hrs or 24 hours. (top) Real-time PCR for β5 demonstrates no increase in β5 gene expression. (bottom) Western blot for uPA confirms uPA knockdown. (B) Plasmin activity: HCFs were transfected with non-cleavable uPAR (NC) or control vector (C). NC-uPAR transfected cells were either untreated (−) or incubated with α-2-anti-plasmin (AP). (C–E) HCFs were transfected with either uPA siRNA or control siRNA. (C) Internalization Assay: HCFs were cell-surface biotinylated at 4°C. Cells were warmed to 37°C for 30 minutes and the remaining cell-surface biotin was cleaved. Cells were lysed and biotin-containing proteins were precipitated with streptavidin beads. Integrin β5 was detected by Western blot. (D) Degradation Assay: HCFs were cell-surface biotinylated at 4°C. Cells were warmed to 37°C for the indicated time points before lysing. Biotin-containing proteins were precipitated with streptavaidin beads. Integrin β5 was detected by Western blot. Numbers from densitometry of β5 bands were equalized to GAPDH and graphed as percent remaining compared to time zero. (E) Ubiquitination of β5: HCFs were IPed with antibody to ubiquitin (mono or poly ubiquitin, FK2 antibody) and integrin β5 was detected by Western blot. F. HCFs were transfected with siRNA to tsg101 and hrs ESCRT subunits. After 48 hours cells were lysed and Western blotted for β5. GAPDH indicates equal loading (A, B, and F) or equal protein input for IP (C and E). Knock down of hrs and tsg101 together (ESCRT) was validated by RT-PCR. β5 expression after ESCRT knockdown is also shown. N = 3 for each experiment.

To determine whether uPA exerts transcriptional control on integrin β5 gene expression, RNA extracted from uPA-silenced cells and controls at 6 and 24 hrs was evaluated by real time PCR; β5 expression is not increased after uPA-silencing (Figure 3A). These data indicate that the increase in β5 protein levels is due to translational or post-translational control.

Human corneal cells in serum-free medium, in which our experiments are performed, do not produce plasminogen [23]. However, since the HCFs are passaged in serum-containing medium (with plasminogen), and since plasminogen binds to the cell-surface, we wanted to exclude the possibility that αvβ5 was increased after uPA-silencing only because uPA-generated plasmin on the cell-surface was not present to degrade integrin β5. To test this, cell-surface uPA activity was increased with non-cleavable uPAR (as in Figure 2) and then plasmin was blocked with α-2-anti-plasmin (AP) [24]. As expected, NC-uPAR over-expression led to a reduction in β5 compared to control (Figure 3B, lanes 1 and 3). Treating cells with α-2-anti-plasmin did not affect the protein levels of β5 suggesting that plasmin is not involved in the observed decrease of this integrin (Figure 3B, lane 2). Furthermore, it is unlikely that uPA is directly degrading β5. There were no β5-derived cleavage products on Western blots (probed with a polyclonal anti-β5 antibody) as has been reported for uPA-mediated cleavage of integrin α6 [25]. In addition, treatment with uPA did not produce any β5 degradation products, even if the cells were first stripped of endogenous uPA (data not shown).

Since cell-surface β5 was increased after uPA-silencing (Figure 2), we tested whether uPA-silencing reduced β5 internalization. To address this question, HCFs were transfected with uPA or control siRNA and after 24 hrs cell-surface biotinylated at 4°C and then warmed to 37°C to stimulate endocytosis. After 30 minutes, the remaining cell-surface biotin was cleaved. Cells were then lysed and immunoprecipitated with streptavidin beads and Western blotted for β5. Contrary to expectation, we found an average 2.5-fold increase in internalized β5 in uPA-silenced cells than in control cells (Figure 3C), suggesting that inhibition of β5 internalization does not contribute to the accumulation of β5. The build-up of β5 (cell-surface and total) upon uPA-silencing, which occurs without a defect in β5 internalization, suggests that internalized β5 is not being degraded and may continue to be recycled back to the cell-surface. Thus, we asked if the degradation rate of internalized β5 was affected by uPA-silencing. HCFs were transfected with uPA siRNA or control siRNA and after 24 hours the cells were cell-surface biotinylated for 30 minutes at 4°C and then returned to 37°C for the indicated times. Strikingly, we found that compared to control siRNA, β5 degradation was inhibited by an average of 66% in uPA-silenced cells at 24 hrs (Figure 3D). This time scale for degradation of integrins is similar to what has been previously described [26]. Because of the considerable reduction in the β5 degradation rate, we asked whether uPA-silencing reduced β5 ubiquitination. In Figure 3E, we show that there is an average 56% decrease in ubiquitin labeling of β5 after uPA-silencing, suggesting that it is an important contributor to β5 accumulation. Finally, to further confirm that β5 ubiquitination influences the levels of β5 and to determine if integrin β5 is degraded in the endosomal pathway, we transfected siRNAs to endosomal ESCRT proteins, tsg101 and hrs. ESCRT proteins play a role in sorting ubiquitinated proteins through the endosomal pathway [27]. Depletion of these two ESCRT subunits produces more dramatic effects on the accumulation of ubiquitinated proteins in the endosomal pathway than a single depletion by preventing cargo from reaching the lysosome for degradation [28]. In Figure 3F we demonstrate that simultaneous silencing of tsg101 and hrs results in a 2.4 fold increase in the levels of integrin β5. An 85% knock down of tsg101 and 42% knockdown of hrs was confirmed by RT-PCR. Furthermore, we confirmed that β5 gene expression was not increased by ESCRT knockdown even though protein levels are elevated. Together these data show that uPA regulates β5 ubiquitination and that β5 ubiquitination is necessary for its processing through the endosomal pathway.

### uPA deficiency has Mfs characteristics; increased cell area and α-SMA stress fiber organization

**Figure 4:**
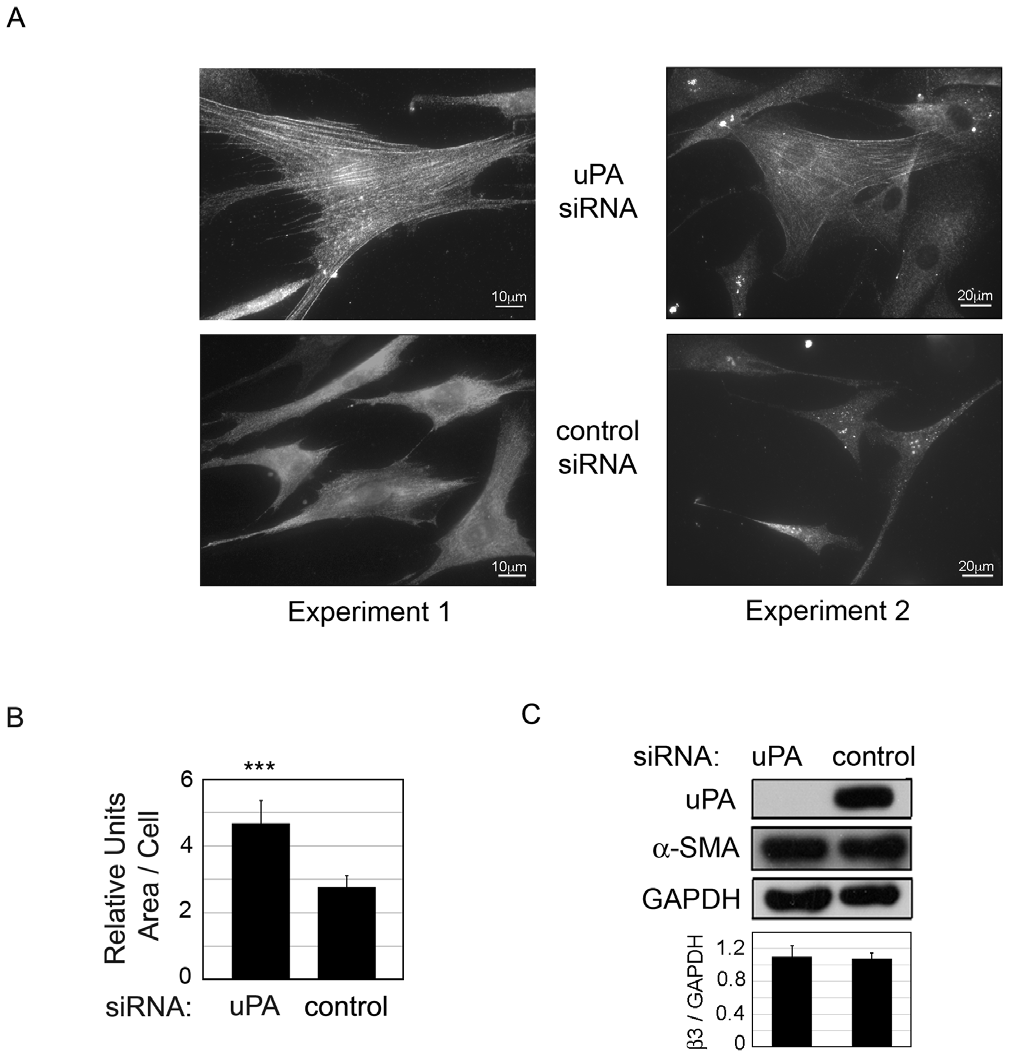
siRNA knockdown of uPA triggers incorporation of α-SMA into stress fibers. HCF were transfected with siRNA against uPA or control siRNA and after 24 hours (A) cells were immunostained for α-SMA. Bar = 10 um or 20 um. Images are from two independent experiments. (B) Metamorph analysis quantified changes in average cell size in each condition. ***p value<0.001. (C) Cell lysates were Western blotted for α-SMA and for uPA to confirm knock down. GAPDH indicates equal loading. Densitometry for α-SMA/GAPDH is shown. N = 3–5 for each experiment.

As previously stated, downregulation of uPAR (and therefore uPA/uPAR binding) and increased β5 is associated with the Mf phenotype, suggesting that uPA/uPAR-mediated control of β5 may play a role in Mf differentiation. Although the primary ligand of αvβ5 is VN, *in vivo*, collagen is a major constituent of fibrotic tissue. For this reason, all of our experiments were performed on collagen. To determine if uPA-silencing promoted fibrotic phenotypic changes, uPA-silenced were immunostained for α-SMA containing stress fibers, a key marker of Mf differentiation. In Figure 4A, we demonstrate that uPA-silencing generates large cells, and approximately 19%+/−5% of HCFs transfected with uPA siRNA had immunodetectable α-SMA in stress fibers whereas in control HCFs there were none. (Images from two separate experiments are shown). Metamorph analysis showed the area of uPA-deficient cells was 1.7-fold larger on average than control cells (Figure 4B). Parallel studies performed with uPAR siRNA showed similar but a slightly lesser increase in cell area (1.4-fold +/−0.14, ***p value = <.001, data not shown).

Earlier studies reported that αvβ5 on Mfs plays a role in activating TGFβ [13], [15], [29]. Since we detected elevated levels of β5 after uPA-silencing we investigated whether this was accompanied by an increase in TGFβ activity. Using a luciferase bioassay [30] and an ELISA assay for active TGFβ, we were surprised to find that there was no difference in the amount of active TGFβ between uPA-silenced cells and control (24 hrs after transfection) (data not shown). (This was also true when the cells were assayed in serum-containing medium.) Western blots showed approximately equal expression of α-SMA in uPA-silenced cells versus control (Figure 4C) but only uPA-silencing generated enlarged cells with α-SMA organized in stress fibers. Together our data suggest that after uPA-silencing the cells exhibit increased cell/matrix tension, which induces cell spreading and α-SMA stress fiber organization without further increase in active TGFβ. Wipff et al. demonstrated that Mf-generated mechanical tension induces integrin β5-mediated activation of TGFβ even in the presence of protease inhibitors [15]. However it is unknown what the role of cell surface uPA protein, uPA/uPAR binding, and uPA/integrin β5 binding [31], [32] is in this type of TGFβ activation. One interpretation of our data is that αvβ5-mediated TGFβ activation requires uPA. Next we used a αvβ5 blocking antibody to determine if the phenotype changes induced by uPA-silencing are mediated by αvβ5.

### αvβ5 blocking antibody reverses the phenotypic effects of uPA siRNA

**Figure 5:**
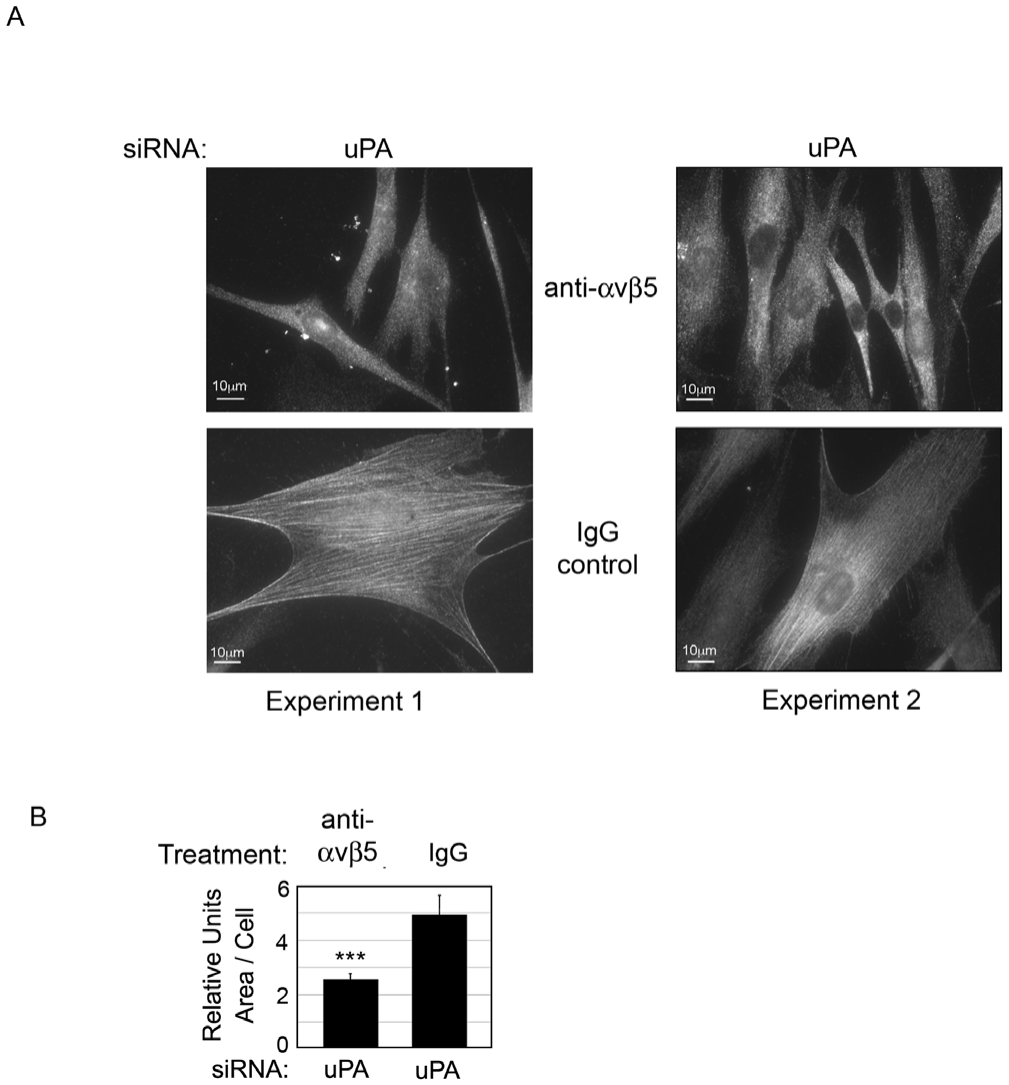
Treatment with anti-αvβ5 antibody reverses the effect of uPA-silencing. HCFs were transfected with uPA siRNA and seeded with a blocking αvβ5 antibody or matched IgG. After 24 hours (A) cells were immunostained with anti-α-SMA antibody. Bar = 10 um. Images are from two independent experiments. (B) Metamorph analysis quantified changes in average cell size in each condition. ***p value<0.001. N = 3 for each experiment.

If the increase in cell area, α-SMA organization into stress fibers observed after uPA-silencing in Figure 4 are due to an increase in integrin β5 levels, we would expect these to be eliminated by blocking αvβ5 function. uPA-silenced cells were treated with blocking αvβ5 antibody or matched IgG control. Figure 5A shows that, compared to control IgG, the αvβ5 blocking antibody prevented the enlargement of the cell area and formation of α-SMA stress fibers in uPA-silenced cells. (Images from two experiments are shown). Metamorph analysis demonstrated that αvβ5-blocked cells were on average 50% smaller than control IgG treated cells (Figure 5B), and similar to the non-uPA-silenced cells (Figure 4B).

### Integrin β1 expression levels and activity are mediated by uPA

**Figure 6:**
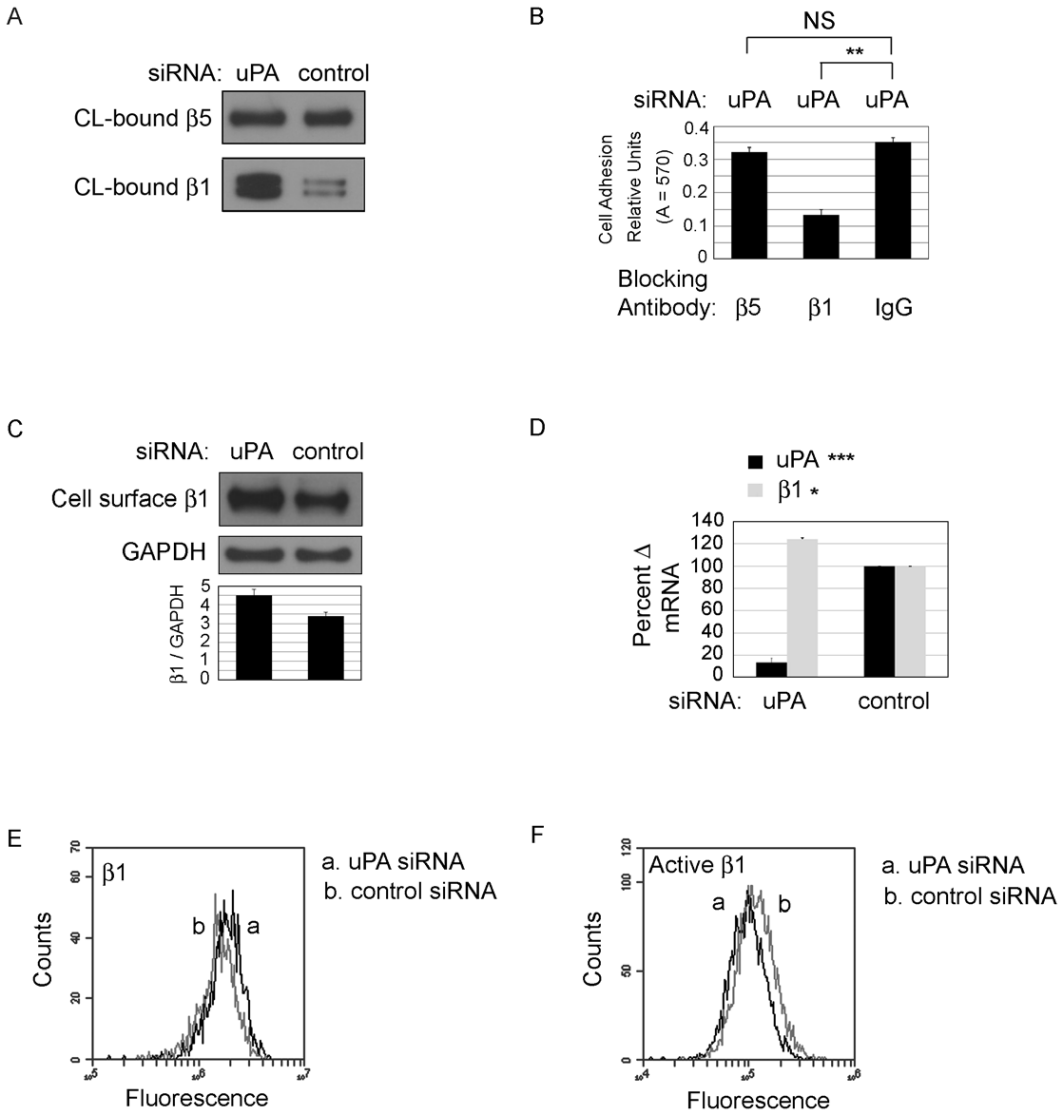
Cell-surface integrin β1 binding to collagen is increased after uPA-silencing but β1 activation is decreased. (A) HCFs were transfected with uPA siRNA or control siRNA. After 24 hours HCF were crosslinked to collagen. After cells were removed with 0.1% SDS, matrix-bound cross-linked proteins were released and equal amounts of protein were analyzed for integrins β5 and β1 by Western blot. (B) Cell adhesion: HCFs were transfected with uPA siRNA. After 24 hours cells were detached and seeded with blocking antibody to β5, β1 or control IgG. After 1 hour, adherent cells were quantified. **p value<0.01. (C–F) HCFs were transfected with either uPA siRNA or control siRNA and cells were analyzed after 24 hours. (C) Cell-surface biotinylation for integrin β1.(D) RT-PCR for uPA and integrin β1, *p value<0.05 and ***p value<0.001. (E) Flow cytometry for integrin β1. (F) Flow cytometry for activated integrin β1. N = 3–5 for each experiment.

Since β5 integrin is a VN-binding integrin we asked whether the cells expressed VN when seeded on collagen. We found that in defined serum free culture, negligible amounts of VN were expressed (Figure S1A). In contrast fibronectin, another αvβ5 ligand, was expressed (Figure S1B). To understand how integrin β5 could promote an increase in cell area and cell tension on collagen we tested whether increased cellular integrin β5 levels were detected as an increase in integrin β5 binding to collagen/fibronectin as we found for β5 binding on VN by uPA silenced cells (Figure 2G). HCFs were transfected with uPA siRNA or control siRNA and seeded on collagen. After 24 hours in culture, a reversible cross-linker was added. Cells were removed with 0.1% SDS and proteins that were cross-linked to the matrix were released. Equal amounts of released protein were Western blotted for integrin β5 (Figure 6A, top). Although β5 binds to matrix (collagen plus secreted fibronectin), the amount of β5 binding was not increased after uPA-silencing suggesting that the increase in cell-surface β5 is not directly responsible for promoting a Mf-like phenotype on this collagen/fibronectin matrix. We next investigated whether integrin β1 subunit, common to many collagen/fibronectin binding integrins contributed to the adhesion and enlarged cell morphology when HCFs were seeded on collagen after uPA-silencing. Unlike integrin β5, we found significantly increased matrix (collagen/fibronectin) binding by integrin β1 after uPA-silencing. These data are supported by our adhesion assay in which initial adhesion of cells transfected with uPA siRNA or control siRNA was tested. After 24 hrs, transfected cells were resuspended and seeded in the presence of blocking antibodies for 1 hour. β5 blocking antibody did not block adhesion on collagen but β1 blocking antibody resulted in a 58% percent decrease in cell adhesion (Figure 6B). The effect of blocking antibodies was identical to uPA-silenced cells for cells transfected with control siRNA (data not shown). A second set of experiments tested the impact of the integrin blocking antibodies on cell attachment after overnight incubation with antibody to parallel the experiment performed in Figure 5A. In this experiment, blocking antibody to either β5 or β1 was added with cells immediately after transfection with uPA siRNA or control siRNA. After 24 hours in culture, cells were washed and adherent cells were quantified. As above, β5 blocking antibody did not alter cell adhesion, and β1 blocking antibody treatment led to a 38% decrease in cell adhesion (Figure S2A). The greater blocking that is observed after a 1 hour treatment compared to blocking overnight is likely because treatment overnight permits non-β1 cell adhesion molecules to recover cell attachment. Consistent with a role for cell spreading, treatment with blocking β1 antibody also resulted in a decrease in cell size (of the remaining attached cells), which were devoid of organized of α-SMA stress fibers that is typically visualized after uPA-silencing (Figure S2B).

We next determined if the cell-surface levels of β1 were affected by uPA-silencing. Using cell surface biotinylation, we found an average 1.33 fold increase in the levels of cell-surface integrin β1 after uPA-silencing (Figure 6C). Unlike β5, this corresponded with an average 1.25 fold increase in β1 gene expression (Figure 6D). The cell-surface levels of β1 were also assessed by flow cytometry in which there was an average 1.24+/−.07 fold increase in integrin β1 cell-surface levels, *p value<.05, after uPA-silencing (Figure 6E). To determine if the increase in β1-collagen binding correlated with a change in β1 activity, flow cytometry was performed using the HUTS-4 antibody that detects the active conformation of integrin β1. We demonstrate that after uPA-silencing, active β1 is decreased by an average 20%+/−.05%, *p value<.05 (Figure 6F). To summarize, Figures 4, 5, and 6 demonstrate that when HCFs are plated on collagen, uPA-silencing induces a Mf-like phenotype and an increase in cell-surface β5, and that although β5 blocking antibody does not detach cells it reverses the Mf-like phenotype, suggesting that integrin β5 is affecting the activity of a β1 integrin.

### Integrin β5 regulates integrin β1

To test if the increase in β5 cell-surface levels after uPA-silencing altered integrin β1 activity, we incubated uPA-silenced cells with β5 blocking antibody overnight as in Figure 5.

**Figure 7:**
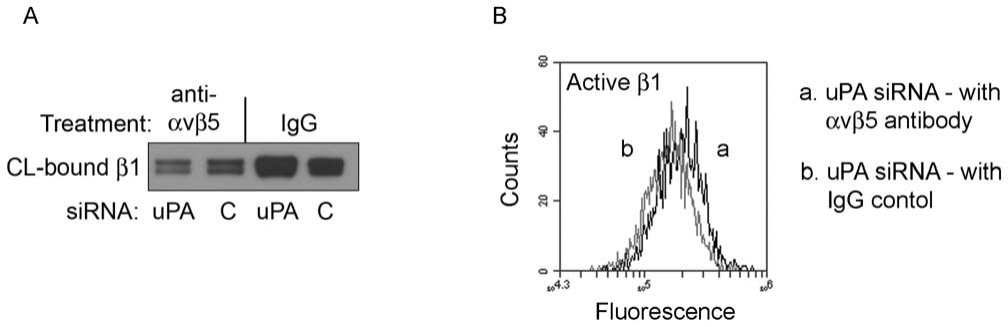
β1 integrin is activated by blocking integrin β5. (A) HCFs were transfected with uPA siRNA or control siRNA and seeded with a blocking αvβ5 antibody or matched IgG. After 24 hours HCF were crosslinked to collagen. The amount of β1 released from the matrix after cell removal is visualized by Western blot. (B) HCFs were transfected with uPA siRNA. After 24 hours, cells were analyzed by flow cytometry for activated integrin β1. N = 3 for each experiment.

Again utilizing the matrix cross-linking assay (Figure 6A), we demonstrate that treatment with αvβ5 blocking antibody reduces integrin β1/matrix binding compared to control IgG (Figure 7A, compare lanes 1 and 3) revealing that integrin β5 mediates β1/matrix binding under these conditions. We also confirm the finding in Figure 6A that uPA-silencing increases β1 binding to matrix by demonstrating that there is increased β1 after uPA-silencing compared to control siRNA when treated with control IgG (Figure 7A, compare lanes 3 and 4). Flow cytometry was used to demonstrate that β5 blocking antibody also increased β1 activation by 1.19+/−.04 fold, *p value<.05 (Figure 7B). Together our data suggest that the increase in β5 cell-surface levels after uPA-silencing promotes β1-mediated cell attachment that correlates with a decrease in integrin β1 activity. Furthermore, this integrin β1-mediated cell attachment to the collagen/fibronectin matrix results in increased cell spreading and α-SMA organization.

## Discussion

Since the persistence of Mfs in wounded tissue correlates with fibrotic disease, uncovering mechanisms that promote Mf differentiation is important to our fundamental understanding and treatment of fibrosis. In the current study, we demonstrated that the absence of uPA led to an increase in total and cell-surface integrin β5, as a result of decreased degradation of internalized αvβ5. The enhanced levels of integrin β5, increased integrin β1 binding to a collagen/fibronectin matrix, generating enlarged cells, which had α-SMA organized into stress fibers.

### Our working model

Our current working model is based on the biologically relevant finding that uPAR is downregulated on Mfs [6]. We have utilized uPA-silencing (and uPAR-silencing) to reveal potential uPA/uPAR-based mechanisms that may be aberrant in fibrotic disease. Here we show that there is a reciprocal relationship between uPA/uPAR and αvβ5. Previous studies demonstrate that over-expression of integrin αvβ5 promotes the Mf phenotype and high levels of αvβ5 are found in fibrotic, sclerodermal tissue [14]. These data together with our findings suggest that the downregulation of uPA or uPAR or a change in uPA/uPAR binding could promote a pathology-causing accumulation of integrin αvβ5 *in vivo*. This hypothesis is supported by an *in vivo* study where in uPAR-deficient mice dermal wound healing was fibrotic as characterized by increased dermal thickness, collagen deposition, and Mf differentiation [33]. Similarly, knocking out uPAR increased the progression of liver fibrosis [34]. Although the observed increase in collagen deposition in these studies is due at least in part to the decreased cell-surface uPA activity, the mechanism responsible for generating Mfs was not determined. Our model predicts that after uPAR knockdown, an increase in integrin β5 contributes to the generation of Mfs in healing wounds. Other studies of fibrotic diseases have discovered alterations in the uPA/uPAR interaction. These include a Scleroderma (SSc) study using vascular endothelial cells in which uPAR expression is upregulated but uPAR is cleaved, thus preventing uPA binding [35], [36]. The authors suggested that reduced uPA/uPAR binding inhibits tissue vascularization leading to ischemia that is a hallmark of SSc. Another study characterized the uPA/uPAR system on fibroblasts from limited and diffuse types of SSc by comparing affected versus non-affected skin in these patients. The ratio of uPA secretion to uPAR expression was increased in the affected areas (1.4 fold for limited SSc and 3.5 fold for diffuse) [37], again suggesting that a change in uPA/uPAR binding is associated with fibrosis. Finally, the importance of uPA/uPAR binding was highlighted in a study in which endogenous uPA/uPAR binding was abrogated in a mouse model, resulting in chronic inflammation secondary to fibrin deposition, both of which are associated with fibrotic disease [38], [39].

### Ubiquitination of integrins and integrin binding to ECM

Accumulation of specific matrix molecules is a hallmark of fibrosis. Since integrins and ECM are internalized together [40], the finding of reduced αvβ5 degradation suggests a parallel reduced ECM degradation and increased recycling of non-degraded integrin/ECM to the cell-surface. A recent study reported that internalized α5β1 is ubiquitinated and targeted to lysosomes for degradation and that fibronectin binding is necessary for α5β1 ubiquitination and degradation of the integrin/ECM complex [26]. The authors proposed that in the absence of degradation, the integrin/ECM complex would be recycled to the cell-surface, leading to an increase in cell adhesion and a build-up of ECM, that would lead to stabilized cell adhesion and reduced cell migration. Our data support this model as decreased αvβ5 degradation led to an increase in the cell-surface accumulation of integrin β5. When cells were seeded on VN, the increase in integrin β5 promoted β5/VN binding and cell adhesion (2.4 fold increase) (Figure 2G). In addition, the elevated cell-surface levels of integrin β5 promoted integrin β1/collagen binding (Figures 6 and 7), resulting in enlarged cells and organization of α-SMA containing stress fibers. Both VN and collagen matrices include fibronectin secreted by HCFs even under serum-free conditions (Figure S1). Since the *in vivo* fibrotic matrix includes VN, collagen and fibronectin, it is possible that a change in uPA/uPAR status that produces an increase in integrin β5 cell-surface levels, could generate Mf-like cells on VN directly and/or induce integrin β1 binding to a collagen/fibronectin matrix. (Integrin cross-talk is discussed below.) Furthermore, since VN and αvβ5 are internalized together [41], we propose that the accumulation of αvβ5 could lead to an accumulation of VN in the ECM that is associated with fibrotic healing [42].

When considering how uPA-silencing alters ubiquitination of integrin β5, one possibility is that the binding of uPA to integrin αvβ5 [31], [32] may change the conformation and/or phosphorylation state of αvβ5 and make it accessible to ubiquitination. Previous studies demonstrate that phosphorylation of integrins is necessary for a wide range of integrin functions such as migration, adhesion, and downstream signaling [43], [44], [45]. Furthermore, dependence for ubiquitination on the phosphorylation state of the focal adhesion component, talin has been described [46]. Alternatively, since the uPA inhibitor, PAI-1 stimulates the internalization of uPA/uPAR with the LRP receptor and integrins αvβ3 and αvβ5 [47], [48], perhaps, αvβ5 internalization through an uPA-mediated pathway is necessary for αvβ5 ubiquitination and degradation.

### Crosstalk between integrins

Crosstalk between integrins or between integrins and other adhesive molecules such as cadherins, plays an important role in controlling cellular activities such as cell migration, cell adhesion, and ECM remodeling [49]. Specific to our data are studies demonstrating crosstalk between αvβ5 and the collagen binding integrin, α2β1, which report that α2β1 function (stimulation or repression) is dependent on the levels of integrin αvβ5. Utilizing human colon cancer cells lines, the authors found that cell-surface αvβ5 repressed α2β1-mediated cell migration on collagen, which could be overcome by treatment with a β1-activating antibody (TS2/16). In contrast, blocking αvβ5 increased α2β1-mediated cell migration (approximately 5-fold) and addition of β1 activating antibody did not further increase cell migration, suggesting that under conditions in which αvβ5 is blocked, β1 is maximally activated [50]. These data correlate with our findings that high levels of β5 increase β1 binding to collagen/fibronectin matrix (stabilized cell adhesion, in line with reduced cell migration) with reduced β1 activity (Figure 6), whereas blocking antibody to αvβ5 reduced β1 binding to this matrix and increased β1 activity (in line with increased cell migration) (Figure 7). Future studies will define which β1 integrin is activated by αvβ5 on Mfs. In addition to α2β1, α11β1, has been demonstrated to be a key mechanical sensor that influences Mf differentiation on collagen [51] and thus it may be regulated by αvβ5.

### Accumulation of αvβ5 and TGFβ

Our data suggest that together, a TGFβ-mediated increase in β5 gene expression (Figure 1C) and a decrease of uPA/uPAR on Mfs (Figure 1C and [6]), could lead to a pathology-promoting accumulation of αvβ5 and could result in an αvβ5-mediated autocrine loop of TGFβ activation. Although, we did not observe the expected increase in active TGFβ after uPA-silencing (as discussed in the results section), the profound changes in cell size and the generation of α-SMA containing stress fibers, in cells that had none, without observable differences in TGFβ activity revealed that these changes were not TGFβ-dependent. Furthermore, the finding that α-SMA expression was not altered after uPA-silencing (as would be expected without an increase in TGFβ activity) suggests that the change in cell size and incorporation of α-SMA into stress fibers after uPA-silencing is primarily the result of enhanced mechanical tension, which, in the presence of α-SMA, generates its incorporation into stress fibers. These data are consistent with reports that the generation of mechanical tension is a key to the persistence of Mfs [11], [51], [52] and supports the idea that although TGFβ is a primary inducer of ECM accumulation and fibrosis, that targeting adhesion molecules such as integrins, in order to reduce persistent (adherent) Mfs may produce successful anti-fibrotic therapies [53]. Towards this end, blocking the epithelial specific αvβ6 integrin has proven successful in animal models for reducing fibrotic disease [54], [55].

### Conclusion

In summary, uPA-silencing provides a useful model to study the effects of increases in cell adhesion and mechanical tension that lead to α-SMA stress fiber organization without invoking additional effects of high levels of TGFβ. Based on our data potential therapeutic targets to combat fibrosis may be maintaining uPA bound to uPAR on the cell-surface by preventing uPAR cleavage. The downstream signaling that results from inhibiting uPAR cleavage is under investigation [56], [57]. In addition, directly blocking integrin αvβ5 or targeting the ubiquitin pathway to increase the rate of integrin αvβ5 degradation may generate new therapies to reduce fibrotic healing.

## Materials and Methods

### Antibodies and reagents

Integrin β5 antibody for blocking and flow cytometry was from R & D Systems (MAB2528). Integrin β5 antibody for Western blot and IP (ab15459), uPA antibody (Western blot) (ab24121), and GAPDH antibody (ab36845) were from Abcam. α-SMA antibody (04–1094) and ubiquitin antibody (FK2) were from Millipore (04–263). uPAR antibody (R3) (BPDMONR3) was from Enzo Life Sciences. Integrin β1 antibody for flow cytometry was from R&D Systems (MAB17781). Antibody to active form of β1 (HUTS-4, MAB2079Z) was from Millipore. Secondary Alexa-488 and Alexa-568 were from Molecular Probes, Eugene, Oregon. HRP-conjugated anti-Streptavidin antibody and all HRP-conjugated secondary antibodies were from Jackson Laboratories. Magnetic Streptavidin beads were from Pierce. Human siRNAs to uPA uPAR, tsg101, and hrs was from Santa Cruz Biotechnologies. The non-targeting fluorescent nucleotide control (siGlo) was from Dharmacon. Bovine Collagen type I (PureCol) was from Inamed. α-2-anti-plasmin was from Abcam (ab77936). The uPAR mutant construct (non-cleavable uPAR) was a generous gift of Dr. Lilliana Ossowski, Mount Sinai School of Medicine, NY, NY. The construction of this vector was described previously [22].

### Cells and media

Human primary corneal fibroblasts (HCF) were derived from the stroma of human corneas that were not suitable for transplantation (obtained from NDRI, Pittsburgh, PA). Stromal fibroblasts were isolated as previously described [6] and maintained in complete media: DMEM-F12 (Invitrogen) with 10% FBS (Atlanta Biologicals) with ABAM and Gentamicin (Sigma). For experiments except where noted cells were plated on 10 ug/ml collagen in supplemented serum-free media (SSFM): DMEM-F12 plus 1× RPMI-1640 Vitamin Mix, 1× ITS Liquid media supplement, 1 µg/ml glutathione; (all from Sigma), 2 mM L-glutamine, 1 mM sodium pyruvate, 0.1 mM MEM non-essential amino acids; (Invitrogen) with ABAM and Gentamicin.

### Immunocytochemistry

Cells were fixed with 3% p-formaldehyde (Fisher Scientific, Fair Lawn, NJ) and permeabilized with 0.1% Triton X-100 (Sigma). After blocking with 3% normal mouse serum (Jackson Immuno Research), cells were incubated with α-SMA antibody and then secondary antibody labeled with Cy3. Coverslips were viewed with a Zeiss Axioskop microscope and images were captured using a Zeiss Axioscope with a SPOT-2 CCD camera (Diagnostic Instruments, Sterling Heights, Michigan) and processed by Adobe PhotoShop software.

### Western blots

Cells were lysed in Triton buffer (1% Triton, 150 mM NaCl, 20 mM Tris, pH 7.5) plus complete protease inhibitor tablet (Roche) and PMSF (Fisher Scientific). 30 µg of protein was separated on 4–12% NuPAGE gradient gels under reducing conditions (β5, α-SMA, and GAPDH), or non-reducing (uPAR and uPA), and transferred to PVDF membranes. Primary antibody was added to 5% BSA in TBS and secondary antibody was added to 1% milk in TBS. Bands were visualized with ECL (Pierce).

### Blocking αvβ5 with antibody

HCF were seeded on collagen in SSFM and grown for 48 hours with 1 ng/m TGFβ1 before addition of 2.5 ug/ml anti-αvβ5 function blocking antibodies or IgG controls for 24 hours (Figure 1). To block αvβ5 after uPA siRNA transfection, 2.5 ug/ml anti-αvβ5 or matched IgG was added to the transfected cells before seeding on collagen in SSFM (Figure 5).

### Transfections

Transient transfection was performed using the Amaxa Nucleofection® system (Gaithersburg, MD). HCFs were transfected using the NHDF kit with 10 µM of human targeting uPA or uPAR siRNA or 10 µM control siRNA (siGLO) and seeded on collagen in SSFM without antibiotics. Cells were analyzed after 24 hours. For ESCRT protein knockdown, 10 µM hrs plus 75 µM tsg101 [26] were transfected and compared to 85 µM siGLO. Cells were lysed for Western blot after 48 hours. Knockdowns were confirmed by Western blot and RT-PCR. For the over-expression of non-cleavable uPAR, HCF were transfected with 2.0 µg human NC-uPAR cDNA, or control pcDNA in 10% FBS and F12-DMEM without antibiotics. The next day media was changed to SSFM and the following day cells were lysed for Western blotting, or uPA assay (Figure 2). Over-expression was confirmed by RT-PCR. Transfection efficiency (approximately 80%) was monitored by separately expressing EGFP.

### PA activity assay

This assay was performed as previously described [6]. Briefly, cells were plated onto collagen in SSFM experiments were performed as described above. Cells were lysed in 0.1% Triton-X-100 in 100 mM Tris pH 8.0. After equalizing protein concentrations, uPA activity was determined by incubation with 2 ug of plasminogen and generation of plasmin was determined using a chromogenic plasmin substrate (Spectrozyme PL, American Diagnostic). The reaction product was measured on a Biotek spectrophotometer (A405 nm) at 1 hour.

### Plasmin activity inhibition

HCFs were transfected with 2.0 µg human non-cleavable uPAR cDNA, or control pcDNA in 10% FBS and F12-DMEM without antibiotics. The next day media was changed to SSFM and either not treated or treated with 10 ug/ml α-2-anti-plasmin for 24 hours before lysing with Triton buffer plus inhibitors.

### RNA extraction and real time PCR

Total RNA was extracted from cell lysates using the TRI Reagent RT kit (MRC, Cincinnati, OH) and cleaned by RNeasy Mini Elute Cleanup Kit (Qiagen, Valencia, CA). First stranded cDNA was generated from 1 µg of total RNA using the Superscript First Strand and oligo dT (Invitrogen) according to the manufacturer's instructions. Absolute Blue qPCR master mix (Fisher) was used to generate PCR product. Triplicate determinations were analyzed using the ABI 7900 sequence detection system. Annealing temperature was 55°C for all reactions. Primers used were β5 (IDT):CTGTCCATGAAGGATGACTT, TGTCCACTCTGTCTGTGAGA; β3(IDT):GTAGCCAAACATGGGCAAGC, TCACCAGTAACCTGCGGATTG; uPAR(Invitrogen): CTGGAGCTGGTGGAGAAAAG, GCTTCGGGAATAGGTGACAG; GAPDH (Invitrogen):TTGATTTTGGAGGGATCTCG, GAGTCAACGGATTTGGTCGT; uPA (Invitrogen) GGGGAGATGAAGTTTGAGGT, CTCTTTTCCAAAGCCAGTGA. Integrin β1(IDT); CCTTCTATTGCTCACCTTGTCC, ACTTGGGACTTTCAGGGATG, Tsg101(IDT);GGACACATACCCATATAATCCCC, TCATCCGCCATCTCAGTTTG, Hrs (IDT); AGGAGAAGGAGAGGCTGAG, TGTGGTTACTCTTCATGCGG; Vitronectin (IDT); TGCTGGCATGGGTTGCT, GTTCATGGACAGTGGCATTGTT; Fibronectin (IDT); TGATCACATGGACGCCTGC, GAGTCAAGCGGACACAACG.

### Immunoprecipitation (IP)

Figure 1A: Cells were treated for 72 hours with either 1 ng/ml TGFβ1 or 5 ng/ml FGF-2 with 1 ug/ml heparin in SSFM on collagen. For cell-surface expression, cells were biotinylated for 30 minutes with 0.5 mg/ml EZ-Link Sulfo-NHS-LC-Biotin (Pierce). Cells were lysed in Triton Buffer plus protease inhibitors. For each IP, 5 ug of β5 antibody were added to 0.5 mg of total protein and incubated overnight at 4°C. Eluted proteins were separated under reducing conditions. Western blots were processed in 5% BSA in TBS and incubated with streptavidin-HRP. Bands were visualized with ECL (Pierce). Figure 2F: HCFs were transfected with either uPA siRNA or control siRNA. After 24 hours cells were biotinylation as above. Cell lysis in RIPA buffer (50 mM Tris, pH 7.4, 150 mM NaCl, 0.1% SDS, 0.5% Sodium Deoxycholate, 1% Triton) plus inhibitors. Biotinylated proteins were precipitated using streptavidin coated magnet beads (MagnaBind, Thermo Scientific). The proteins were eluted under reducing conditions and Western Blotted for integrin β5. Figure 3E: HCFs were transfected with either uPA siRNA or control siRNA. After 24 hours cells were lysed with RIPA plus protease inhibitors, IPed with antibody to ubiquitin (FK2) and immunoprecipitates were Western blotted for β5.

### Adhesion Assay

Figure 2G: HCF were transfected with either uPA siRNA or control siRNA as above. The next day, cells were resuspended with Detachment buffer (enzyme-free cell dissociation solution, Millipore) and 20,000 cells were seeded in SSFM into vitronectin-coated wells (2 ug/ml). After 24 hours, the cells were fixed with 3% paraformaldehyde and stained with DAPI. Three photos were taken from different areas for each well. The imaged cells were counted automatically using ImageJ “Analyze Particles” (particle size were set from 20 to infinity). The numbers of 3 photos from each well were averaged as the final number for that well. 6 wells were analyzed for each transfection. Figure 6B: HCF were transfected with uPA siRNA or control siRNA. After 24 hours, cells were detached with detachment buffer and seeded onto collagen in a 96 well plate with blocking antibodies: 1.25 ug/mL β5 antibody, 1.25 ug/mL β1 antibody or 1.25 ug/mL control IgG. After 1 hour cells were washed with PBS thoroughly with tapping and fixed by methanol and stained with 0.1% crystal violet for 1 hour at room temperature. Crystal violet was eluted with 10% acetic acid and the eluate was measured at 570 nm. Figure S2A: HCF were transfected with uPA siRNA or control siRNA and 25,000 cells were seeded onto collagen in 96-well plates with blocking antibodies: 1.25 ug/mL β5 antibody, 1.25 ug/mL β1 antibody or 1.25 ug/mL control IgG. After 24 hours cells were washed and stained with cystal violet and measured as above.

### Cross-linking Assay

HCF were transfected with uPA siRNA and control siRNA. Cells were seeded on collagen in SSFM on a 100 mm dish. The next day cells and matrix were cross-linked by 1 mM BSOCOES (Thermo Scientific) for 10 minutes, and then quenched twice with a buffer containing (50 mM Tris-HCl (pH 7.2) and 100 mM NaCl). The cells were then removed by lysis with 0.1% SDS with protease inhibitors and the plates were washed three times with PBS. After washing, the plates were incubated in 50 mM NaHCO_3_ (pH 11.6) and 0.1% SDS with protease inhibitors at 37°C with shaking for 2 hours to reverse cross-linking. The soluble fraction was concentrated by Amicon Ultra (Millipore) and stored at −80°C. Equal protein from each sample was analyzed by Western blotting. A lack of GAPDH on Western blots confirmed that cells were not present in the protein fraction that was released from the matrix.

### Internalization Assay

Our method was based on the paper by Vassilieva et al. [58]. HCFs were transfected and cell-surface proteins were biotinylated as above. Labeled cells were gently washed three times in cold PBS and then incubated at 37°C in SSFM for 30 minutes. Biotin remaining on the cell-surface was cleaved with buffer containing reducing agent [100 mM MESNA (sodium-2-mercaptoethane sulfonate), 50 mM Tris (pH 8.6), 100 mM NaCl, 1 mM EDTA, and 0.2% BSA] at 4°C (3 washes). The cells were rinsed twice in ice-cold PBS, and excess biotin was quenched with 60 mM iodoacetamide in PBS for 5 min at 4°C. Cells were then washed three times in ice-cold PBS and lysed in RIPA buffer plus inhibitors. The lysates were clarified by centrifugation at 14,000 g for 10 min. Biotinylated proteins were precipitated using streptavidin coated magnet beads. The proteins were eluted under reducing conditions and Western Blotted for integrin β5.

### Degradation Assay

HCFs were transfected and cell-surface proteins were biotinylated as above. Labeled cells were gently washed twice in cold PBS. Cells were returned to 37°C and at the specified time points (0, 1, 6, 24 hours) cells were quenched by 100 mM glycine and lysed by RIPA buffer plus inhibitors. Biotinylated proteins were precipitated using streptavidin coated magnet beads. The proteins were eluted under reducing conditions and then Western blotted for integrin β5. (Since the cells were incubated for 24 hrs after biotin labeling, to avoid additional perturbation of the cell culture, the cleaving of biotin as in the internalization assay was omitted.)

### Flow cytometry: Detection of Integrin β5, β1 and active β1

Integrin β5: HCFs were treated with primary antibody (R & D systems) at 1.25 ug/mL at 4°C. After 1 hr, cells were washed by PBS three times, then detached by enzyme-free cell dissociation solution (Millipore) and resuspended in PBS with 3% BSA. 4×10^5^ cells/400 ul were removed and incubated with goat anti-mouse Alexa-488 (1∶400, Jackson ImmunoResearch) for 30 min at 4°C. The cells were then washed twice by PBS with 3% BSA and filtered into a 5 mL polystyrene tube with cell-strainer cape (BD Falcon). After being stained by propidium iodide, the cells were analyzed by Accuri C6 flow cytometer. Integrin β1 (cell-surface or active): Cells were detached by enzyme-free cell dissociation solution (Millipore) and resuspended in DMEM with 3% BSA. 1×10^5^ cells/100 ul were removed and incubated with antibody against integrin β1 (2.5 µg/10^5^ cells) or active integrin β1 (5 µg/10^5^ cells) for 30 min at 4°C prior to washing and incubated with goat anti-mouse Alexa-488 (1∶400, Jackson ImmunoResearch) for 30 min at 4°C. Cells were washed and counted as above. Negative control is the omission of primary antibody in each case. Average fold change between samples is calculated by first subtracting the negative from each sample before averaging the mean fluorescence of the experimental samples and the control samples. The fold change is the average of the mean fluorescence of the experimental samples divided by the average of the control samples. Standard error and p values are given for each set of experiments.

### Statistical Analysis

Numerical data are expressed as the mean +/− standard error of 3 to 7 independent experiments. P-values were calculated using the students t-test. *p value<0.05, **p value<0.01, ***p value<0.001.

## Supporting Information

Figure S1 **HCFs under defined conditions, express fibronectin but not vitronectin.** HCFs were transfected with uPA siRNA or control siRNA. After 24 hours RNA was extracted for RT-PCR. (A) vitronectin and (B) fibronectin. No statistically significant changes were demonstrated between samples in either case. The data is not converted into percent to demonstrate that VN is nearly undetectable in HCFs under serum-free contiditons compared to fibronectin. N = 3 for each experiment.
(TIF)

Figure S2 **Antibody to β1 but not β5 blocks cell adhesion.** (A) Cell adhesion: HCFs were transfected with uPA siRNA and seeded with blocking antibody to β5, β1 or control IgG. After 24 hours adherent cells were quantified. **p value<0.01. (B) HCFs were transfected with uPA siRNA or control siRNA and seeded with 2.5 ug/ml blocking anti-β1 antibody or control IgG. Bar = 10 um. Cells were immunostained for α-SMA. N = 3 for each experiment.
(TIF)
