# Supplementary figures and images for: Degradation of Internalized αvβ5 Integrin Is Controlled by uPAR Bound uPA: Effect on β1 Integrin Activity and α-SMA Stress Fiber Assembly
Source: PLoS One. 2012 Mar 21;7(3):e33915. doi: 10.1371/journal.pone.0033915 (PMC3309951; doi:10.1371/journal.pone.0033915)

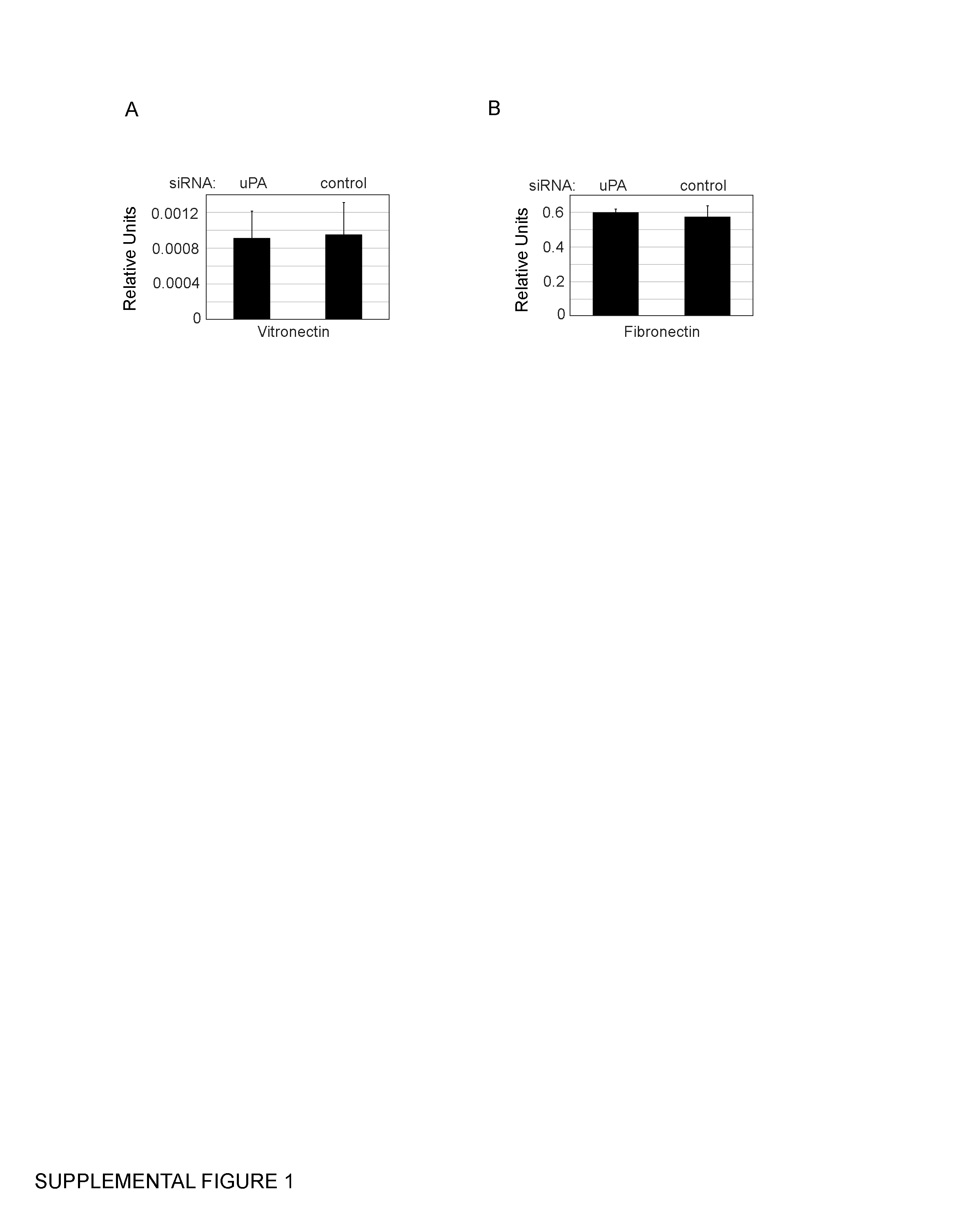

Supplement: Figure S1 — HCFs under defined conditions, express fibronectin but not vitronectin. HCFs were transfected with uPA siRNA or control siRNA. After 24 hours RNA was extracted for RT-PCR. (A) vitronectin and (B) fibronectin. No statistically significant changes were demonstrated between samples in either case. The data is not converted into percent to demonstrate that VN is nearly undetectable in HCFs under serum-free contiditons compared to fibronectin. N = 3 for each experiment. (TIF) [file pone.0033915.s001.tif]

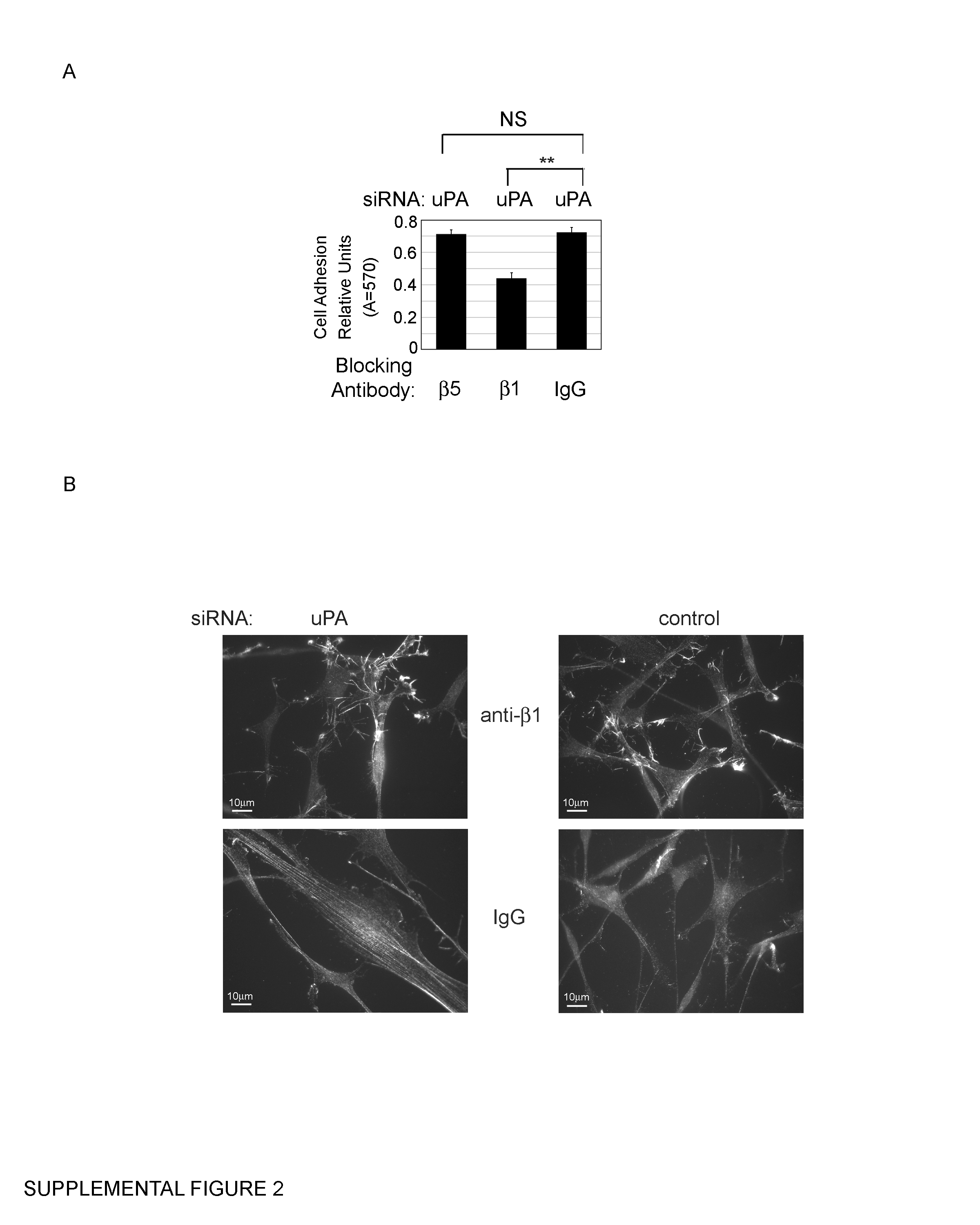

Supplement: Figure S2 — Antibody to β1 but not β5 blocks cell adhesion. (A) Cell adhesion: HCFs were transfected with uPA siRNA and seeded with blocking antibody to β5, β1 or control IgG. After 24 hours adherent cells were quantified. **p value<0.01. (B) HCFs were transfected with uPA siRNA or control siRNA and seeded with 2.5 ug/ml blocking anti-β1 antibody or control IgG. Bar = 10 um. Cells were immunostained for α-SMA. N = 3 for each experiment. (TIF) [file pone.0033915.s002.tif]
